# Tumour response in metastatic renal cell carcinoma treated with tyrosine kinase inhibitors – assessment of intra-tumour heterogeneity

**DOI:** 10.1186/s12916-016-0754-8

**Published:** 2016-12-07

**Authors:** Viktoria Stühler, Jens Bedke

**Affiliations:** Department of Urology, Eberhard Karls University, Hoppe-Seyler- Strasse 3, 72076 Tübingen, Germany

**Keywords:** Metastatic clear cell renal carcinoma, Intra-tumour heterogeneity, Response pattern, Tyrosine kinase treatment, RECIST criteria, Pseudoprogression

## Abstract

Intra-tumour heterogeneity is a common molecular phenomenon in metastatic clear cell renal carcinoma (mRCC), representing the genetic complexity of a tumour with multiple metastatic sites. The present commentary discusses the observed phenomena of phenotypic intra-tumour heterogeneity in mRCC patients treated with the tyrosine kinase inhibitors sunitinib or pazopanib. Here, drug response can be different on the level of each evaluated metastasis in the individual patient. This questions the currently used radiologic staging systems of RECIST criteria and demands for a modification of radiologic response assessment with the consequence of a patient-tailored therapy in the clinical setting.

Please see related article: http://bmcmedicine.biomedcentral.com/articles/10.1186/s12916-016-0729-9.

## Background

Renal cell carcinoma (RCC) is a multifaceted tumour. The same histopathological subtype, stage and grade in clear cell RCC demonstrates a different tumour behaviour among patients, called inter-tumour heterogeneity (ITH) [1]. ITH is a common phenomenon defined by different subpopulations of cells with distinct genomic alterations and phenotypes between the primary tumour and the respective metastases within one patient [2]. Natural selection is the backbone of ITH, leading to an accumulation of genetic alterations in genetically unstable cells through which a selection pressure drives the growth and survival of distinct subpopulations, mirroring a biological fitness advantage. These mechanisms of clonal evaluation and genomic instability of the cancer cell contribute to molecular heterogeneity within the tumours, leading to subclones that are likely to have a growth or survival advantage [3]. The evidence for this genetic diversity both between different tumours and within a single tumour has been derived from new technologies such as next-generation sequencing. Gerlinger et al. [2] revealed extensive ITH by exome sequencing of multiple tumour samples from primary and metastatic lesions in patients with clear cell RCC. Indeed, there is evidence of multiple, genetically distinct subclones within primary tumours or in primary tumours and their metastases [2]. Further, subclonal driver mutations may contribute to the acquisition of drug resistance [4]. This known fact of molecular ITH is likely to influence cancer therapeutics and to result in heterogeneous or mixed response patterns as observed by imaging.

Considerable progress has been made in the treatment of metastatic RCC (mRCC), with an improvement of overall survival following the implementation of anti-angiogenic tyrosine kinase inhibitors (TKIs) since 2006 [5]. Complete response (CR) is a rare event with TKIs; however, partial response (PR) is achieved in 10–39% of patients [6, 7]. In the case of a PR, an additional benefit from surgical resection of residual metastases is observed, achieving prolonged disease control [7, 8]. Nevertheless, the majority of advanced diseases reveal that the first observed clinical benefit is often of limited duration, with most patients exhibiting disease progression [9]. Therefore, the identification of distinct response and progression patterns in the treatment of mRCC is critical. The Response Evaluation Criteria In Solid Tumours (RECIST 1.1 criteria) is the currently accepted method to provide a radiographic definition for CR, PR, stable disease (SD) and progression, and thereby defines progression-free survival time in mRCC [10]. The RECIST method is based on morphologic changes, specifically the change in the sum of the longest dimensions of the target lesions.

### Phenotypic heterogeneity

In a recent article, Crusz et al. [11] hypothesized that the molecular ITH is mirrored by clinical heterogeneity, observed by a subset of metastases responding and progressing within the same patient. In their study, a radiological analysis of patients with two or more assessable metastatic lesions that progressed under therapy with anti-angiogenic TKIs (sunitinib or pazopanib), based on the population of three similar phase II trials, was performed. For the analysis of the study population (*n* = 27 patients with multiple metastases) each metastasis was evaluated based on the principles of RECIST 1.1 to define responding, stable or progressing lesions. A heterogeneous drug response was defined as the deviation of response patterns within one patient, while a homogenous response was defined as all lesions falling within the same response category. Heterogeneous response was detectable in 56% (15/27) of patients and homogenous response in 44%. There was no difference in heterogeneous response in patients who had a suboptimal dosing through dose reductions or those that underwent nephrectomy. Reason for progressions was mainly the appearance of new lesions (67%), while the progression of existing lesions was a rare event (11%); 22% of patients exhibited both.

In clinical practice, the decision to switch or to continue a given systemic therapy is a common challenge, especially in the presence of heterogeneous progression and response patterns. Thus, the identification of cancer types with a respective heterogeneous response pattern is likely to influence clinical decision-making and, therefore, clinical outcome. As shown, a clinical ITH was observed for mRCC upon sunitinib or pazopanib treatment [11]. The occurrence of new lesions, which was the main cause for the definition of progression, questions the applicability of the currently used RECIST 1.1 criteria, particularly considering that progression-free survival, which is one of the main parameters in the assessment of clinical trials, is presently determined by RECIST 1.1 analysis. Currently, the applied therapy is discontinued and alternative treatments are initiated when the patient meets progression-defined parameters by RECIST criteria such as the occurrence of new (small) lesions even if several large lesions remain controlled.

Studies with monoclonal antibodies or cytokines have shown that an increase in total tumour burden, for example, by oedema or inflammation, both of which are defined as progressive disease by WHO and RECIST criteria, is later followed by sustained tumour regression seen as CR, PR and SD. The development of novel criteria, designated as immune-related response criteria, was based on data from ipilimumab clinical trials in melanoma [12]. These criteria incorporate measurable new lesions into total tumour burden and compare this to baseline measurements [12]. Thus, patients are considered to have PR or SD even if new lesions present, provided that the tumour burden of all lesions meets the determined thresholds. According to this definition, if new, small metastatic lesions occur (defined as PD by RECIST) while the bulk of the disease remains controlled, therapy should not be changed, but rather should be continued. The immune-related response criteria acknowledge heterogeneous response patterns, although their underlying principle is an immune-related event defined as pseudoprogression [10] and not, as shown by Crusz et al. [11], of ITH. Likewise, novel imaging approaches like functional imaging, but also the adjustment of current response criteria, which do not automatically consider the appearance of new lesions as a PD, could further assist to detect metastases that differ in their individual biological characteristics. One clinical consequence is a tailored therapy with the resection of the progressing lesion(s), a molecular analysis form biopsy tissue and, if possible, the expansion of systemic therapy to optimise patient care as well as clinical outcome.

## Conclusions

The aim of targeted therapy is to inhibit the critical proliferation or survival pathways on which the tumour depends. This VEGF-based strategy has significantly improved patient outcomes in mRCC within the last years. The underlying tumour biology demonstrates that the majority of somatic gene mutations are not ubiquitously present within a tumour, but is rather characterised by the existence of subclones derived from a common clonal progenitor, with variations between different regions of the same tumour and metastases [2]. As a clinical result, metastatic lesions of the same patients might show heterogeneous progression patterns in radiologic imaging due to different drug sensitivity as reported by Crusz et al. [11]. Currently, the RECIST 1.1 classification is widely used to determine response assessment, but does not acknowledge phenotypic ITH. The clinical consequence in RECIST 1.1-defined progression is the switch to another line of therapy, rather than the continuation of therapy with or without the resection of the resistant lesion. According to molecular findings, systemic therapy could be, if possible, expanded to another active agent. Currently, this is not satisfactorily reflected in radiologic response assessment with the need for modification of radiologic classifications, especially when used in clinical trials of mRCC patients.

## References

[1] Liang YX, He HC, Han ZD, Bi XC, Dai QS, Ye YK, Qin WJ, Zeng GH, Zhu G, Xu CL (2009). CD147 and VEGF expression in advanced renal cell carcinoma and their prognostic value. Cancer Invest.

[2] Gerlinger M, Rowan AJ, Horswell S, Larkin J, Endesfelder D, Gronroos E, Martinez P, Matthews N, Stewart A, Tarpey P (2012). Intratumor heterogeneity and branched evolution revealed by multiregion sequencing. N Engl J Med.

[3] McGranahan N, Burrell RA, Endesfelder D, Novelli MR, Swanton C (2012). Cancer chromosomal instability: therapeutic and diagnostic challenges. EMBO Rep.

[4] Gerlinger M, Swanton C (2010). How Darwinian models inform therapeutic failure initiated by clonal heterogeneity in cancer medicine. Br J Cancer.

[5] Motzer RJ, Hutson TE, Tomczak P, Michaelson MD, Bukowski RM, Rixe O, Oudard S, Negrier S, Szczylik C, Kim ST (2007). Sunitinib versus interferon alfa in metastatic renal-cell carcinoma. N Engl J Med.

[6] Escudier B, Eisen T, Stadler WM, Szczylik C, Oudard S, Siebels M, Negrier S, Chevreau C, Solska E, Desai AA (2007). Sorafenib in advanced clear-cell renal-cell carcinoma. N Engl J Med.

[7] Rini BI, Shaw V, Rosenberg JE, Kim ST, Chen I (2006). Patients with metastatic renal cell carcinoma with long-term disease-free survival after treatment with sunitinib and resection of residual metastases. Clin Genitourin Cancer.

[8] Neill MG, Wei AC, Jewett MA (2007). Consolidative renal cell carcinoma metastatectomy for partial response after multitargeted tyrosine kinase inhibitor therapy. Urology.

[9] Gore ME, Larkin JM (2011). Challenges and opportunities for converting renal cell carcinoma into a chronic disease with targeted therapies. Br J Cancer.

[10] Eisenhauer EA, Therasse P, Bogaerts J, Schwartz LH, Sargent D, Ford R, Dancey J, Arbuck S, Gwyther S, Mooney M (2009). New response evaluation criteria in solid tumours: revised RECIST guideline (version 1.1). Eur J Cancer.

[11] Crusz SM, Tang YZ, Sarker S-J, Prevoo W, Kiyani I, Beltran L, Peters J, Sahdev A, Bex A, Powles T (2016). Heterogeneous response and progression patterns reveal phenotypic heterogeneity of tyrosine kinase inhibitor response in metastatic renal cell carcinoma. BMC Med.

[12] Wolchok JD, Hoos A, O’Day S, Weber JS, Hamid O, Lebbé C, Maio M, Binder M, Bohnsack O, Nichol G (2009). Guidelines for the evaluation of immune therapy activity in solid tumors: immune-related response criteria. Clin Cancer Res.

